# Tumor Necrosis Factor Receptor-Associated Periodic Syndrome: Genetics, Autoinflammation, and Recurrent Pericarditis

**DOI:** 10.1016/j.jaccas.2024.103206

**Published:** 2025-02-12

**Authors:** Ashraf Samhan, Carolyn A. Rasmussen, Carlos E. Prada, Paul C. Cremer, Arthur M. Mandelin, Mohamed Al-Kazaz

**Affiliations:** a Feinberg School of Medicine, Northwestern University, Chicago, Illinois, USA; b Division of Genetics, Genomics and Metabolism, Lurie Children’s Hospital, Chicago, Illinois, USA; c Division of Radiology, Northwestern Memorial Hospital, Chicago, Illinois, USA; d Division of Rheumatology, Northwestern Memorial Hospital, Chicago, Illinois, USA; e Division of Cardiology, Bluhm Cardiovascular Institute, Chicago, Illinois, USA

**Keywords:** autoimmune, chest pain, echocardiography, imaging, pericardial effusion, tachycardia, treatment

## Abstract

**Background:**

Tumor necrosis factor receptor–associated periodic syndrome (TRAPS) is a rare hereditary autoinflammatory disorder caused by mutations in the tumor necrosis factor superfamily member 1A gene, which encodes tumor necrosis factor receptor 1. It typically presents with recurrent fever and other signs of inflammation.

**Case Summary:**

A 26-year-old Hispanic woman presented with recurrent fever, tachycardia, and pleuritic chest pain. She was diagnosed with recurrent pericarditis (RP) secondary to TRAPS. She showed an excellent clinical response upon switching from canakinumab to rilonacept.

**Discussion:**

RP is often labeled idiopathic, but autoinflammatory diseases such as TRAPS should be considered in such cases on the basis of the clinical presentation. Interleukin-1 inhibitors have emerged as promising treatments for RP, with radical pericardiectomy being considered as a last resort.

## Graphical Abstract

**Figure:**
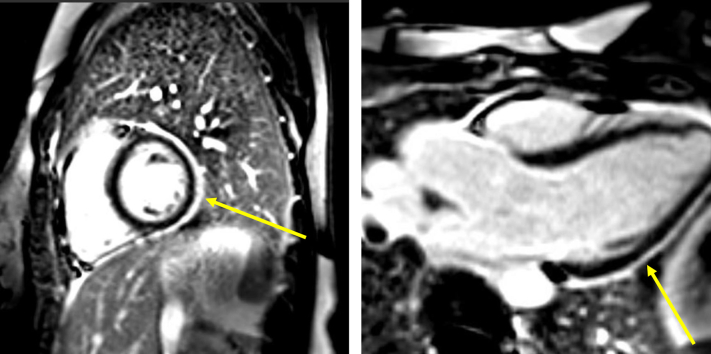

## History of Presentation

A 26-year-old woman presented to the emergency department with recurrent fevers (current episode ongoing for more than 1 week), a 2-day history of pleuritic chest pain radiating to both shoulders, and dyspnea at rest. On physical examination, she was acutely ill appearing, tachycardic, and tachypneic. Cardiac auscultation revealed a pericardial rub. Her vital signs on arrival were the following: temperature 101°F, heart rate 127 beats/min, respiratory rate 20 breaths/min, and blood pressure 100/60 mm Hg.

**Take-Home MessageS**

• Autoinflammation plays a major role in RP.
• Autoinflammatory syndromes should be considered as potential etiologies on the basis of the patient’s profile and clinical course.
• IL-1 inhibitors should be considered in recurrent autoinflammatory pericarditis.
• This case underscores the diagnostic challenges of RP, the connection between TRAPS and RP, and the potential role of IL-1 inhibitors in managing TRAPS-associated RP.

## Past Medical History

The patient’s medical history was significant for a working diagnosis of adult-onset Still’s disease (AOSD), 4 prior episodes of acute pericarditis (attributed to AOSD previously), iron deficiency anemia, and arthralgias. She had been trialed on multiple immunosuppressive therapies for AOSD, including methotrexate, adalimumab, and canakinumab. However, she did not tolerate methotrexate or canakinumab because of gastrointestinal symptoms and experienced recurrent flares despite maximum doses of adalimumab.

The patient is of Hispanic heritage. There is no first-degree family history of periodic fever syndromes or pericarditis. Her biological sister has psoriasis and undifferentiated thyroid disorder, and her mother has psoriasis. Extended family members have histories of autoimmune conditions, including a paternal uncle with myasthenia gravis and a maternal uncle with Crohn’s disease.

## Differential Diagnosis

The differential diagnosis included recurrent pericarditis (RP), myocarditis, AOSD flare, pleuritis, vasculitis, bronchopneumonia, pulmonary embolism, and acute coronary syndrome.

## Investigations

Laboratory values were notable for neutrophil-predominant leukocytosis to 14.3 × 10^9^/L, high-sensitivity troponin < 2 pg/mL, and brain natriuretic peptide 34 pg/mL. C-reactive protein (CRP) and sedimentation rate were elevated to 33.6 mg/mL and 34 mm/h, respectively. Procalcitonin was normal. A respiratory pathogen panel, SARS-CoV-2 nucleic acid test, influenza A/B polymerase chain reaction, and Epstein-Barr virus immunoglobulin M were negative. Blood cultures were negative for infection. Antinuclear antibody was positive at 1:320 titer in a speckled and homogeneous pattern, but the reflexed comprehensive autoimmune disease panel was negative. Serum complement levels were within normal limits. Serum interleukin (IL)–6 was elevated to 69.5 pg/mL. Chest radiography showed no acute cardiopulmonary process. Computed tomographic angiography of the chest revealed a small pericardial effusion but no pulmonary embolism. There was evidence of pericardial thickening but no calcification. Electrocardiography showed sinus tachycardia with nonspecific ST and T-wave changes. Transthoracic echocardiography demonstrated a small circumferential pericardial effusion without tamponade or constrictive physiology (Videos 1 and 2).

## Management

The patient was diagnosed with RP on the basis of clinical presentation, evidence of pericardial effusion on imaging, and elevated inflammatory markers. She was initially treated with intravenous methylprednisolone, colchicine, and ibuprofen. Rheumatology was consulted, and they believed that her symptoms were consistent with an AOSD flare in the setting of being off canakinumab. Notably, she had been treated with colchicine and nonsteroidal anti-inflammatory drugs for previous episodes of pericarditis; however, she could not tolerate them because of gastrointestinal symptoms. Given her intolerance to colchicine, she was discharged with a prednisone taper and canakinumab for a presumed AOSD flare.

**Figure 1:**
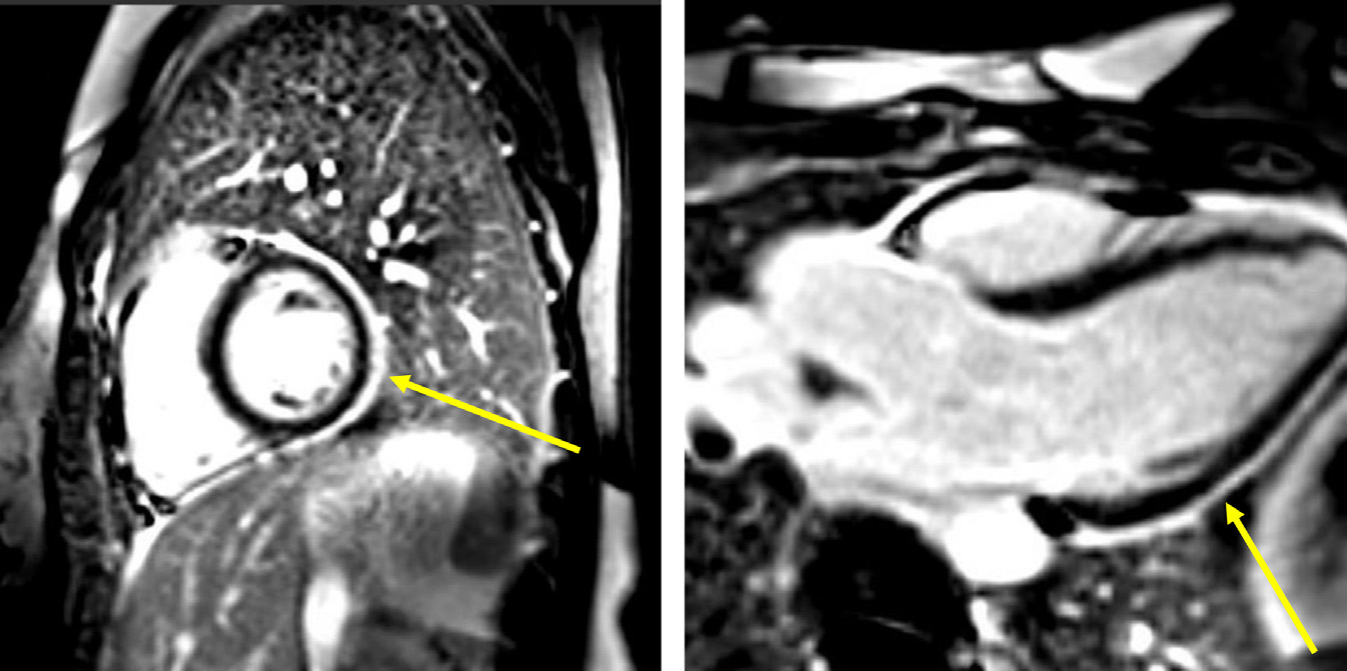
Cardiac Magnetic Resonance Imaging Short-axis (left) and 3-chamber (right) views of cardiac magnetic resonance imaging using a phase-sensitive inversion recovery sequence, showing circumferential pericardial late gadolinium enhancement (yellow arrow).

The patient was referred to the pericardial disease clinic. Her symptoms improved, and CRP had normalized within 3 weeks. Two months after discharge, she experienced daily fevers and pleuritic chest pain despite adherence to her immunosuppressive regimen. Cardiac magnetic resonance imaging was arranged and showed severe pericarditis with 3-mm circumferential pericardial thickening and late gadolinium enhancement (Figure 1). There was no evidence of constrictive physiology.

Given the main presentation of recurrent fevers and pericarditis, the working diagnosis of AOSD was questioned. Furthermore, given her persistent symptoms on canakinumab, the patient was switched to rilonacept with the goal of better controlling autoinflammation with IL-1α and IL-1β blockade^1^ (Table 1).

**Table 1:** Pharmacologic Characteristics of Interleukin-1 Inhibitors^1^^,^^3^

Interleukin-1 Inhibitors for Recurrent Pericarditis		
	Canakinumab	Rilonacept
Mechanism of action	Selective IL-1β	IL-1α = IL-1β > IL-ra
Half-life	∼26 days	∼7 days
Dosing	2-5 mg/kg/month	**Loading** dose: 320 mg once **Maintenance** dose: 160 mg once weekly (given ∼1 week after loading dose)
FDA-approved indications	CAPS Rheumatoid arthritis	CAPS Recurrent pericarditis
Potential side effects	Nasopharyngitis, diarrhea, influenza, headache and nausea (most common), rarely transaminitis, neutropenia, thrombocytopenia	Injection site reaction and upper respiratory tract infections (most common), rarely transaminitis, dyslipidemia, neutropenia
Relevant clinical studies	CLUSTER (Phase 3 trial done by De Benedetti et al^11^)	RHAPSODY (Phase 3 trial done by Klein et al^3^)

CAPS = cryopyrin-associated periodic syndromes; CLUSTER = Canakinumab Pivotal Umbrella Study in Three Hereditary Periodic Fevers; RHAPSODY = Rilonacept Inhibition of Interleukin-1 Alpha and Beta for Recurrent Pericarditis: a Pivotal Symptomatology and Outcomes Study; RCT = randomized control trial.

Because of recurrent periodic fevers with arthralgia and no clear autoimmune disease, the patient underwent genetic testing for monogenic autoinflammatory disorders to evaluate for periodic fever syndromes. She was found to have a heterozygous variant of uncertain significance in tumor necrosis factor superfamily member 1A (TNFRSF1A) (c.418C>A; p.Leu140Ile). Pathogenic variants of this gene are associated with tumor necrosis factor receptor–associated periodic syndrome (TRAPS); however, this specific variant has not yet been reported in the literature in association with the disease. She was referred for genetic counseling, and it was ultimately concluded that the etiology of her pericarditis was most likely secondary to TRAPS.

## Outcome and Follow-Up

Six weeks after initiating rilonacept, the patient reported resolution of her chest pain, and her high-sensitivity CRP had normalized. There are plans to serially monitor her inflammatory markers and repeat cardiac magnetic resonance imaging after 1 year of therapy to guide management. Pericardiectomy will be considered if rilonacept therapy fails in the future or the patient develops an intolerance to it, following shared decision making. She currently does not plan to become pregnant. Genetic testing on her family revealed that the mutation was inherited from her mother.

## Discussion

Acute pericarditis has a wide range of etiologies, including infectious, autoimmune, autoinflammatory, neoplastic, drug-induced, and idiopathic causes. Despite thorough diagnostic efforts, the underlying cause remains unidentified in a significant proportion of cases, leading to the label of “idiopathic” pericarditis. Standard treatment regimens include nonsteroidal anti-inflammatory drugs, colchicine, and/or corticosteroids. However, up to 30% of patients experience recurrent episodes within 18 months of the initial occurrence, necessitating alternative therapeutic approaches.^2^ Recent advances have highlighted the efficacy of IL-1 inhibitors, particularly in cases unresponsive to or intolerant of traditional therapies. This class of medication offers a more effective and targeted approach by modulating the inflammasome and the inflammatory cascade implicated in pericarditis. IL-1, a proinflammatory cytokine, has been implicated in the pathogenesis of RP1. IL-1 inhibitors, such as rilonacept, have demonstrated more rapid resolution of typical pain, quicker normalization of CRP levels, fewer incidences of recurrence, and a greater likelihood of tapering off corticosteroids compared with placebo.^3^ Furthermore, they have been shown to have a favorable safety profile and are generally well tolerated. Cases of canakinumab for the treatment of RP have yielded mixed results.^1^ Further studies are needed to define optimal dosing strategies and their role in different patient populations.

**Figure 2:**
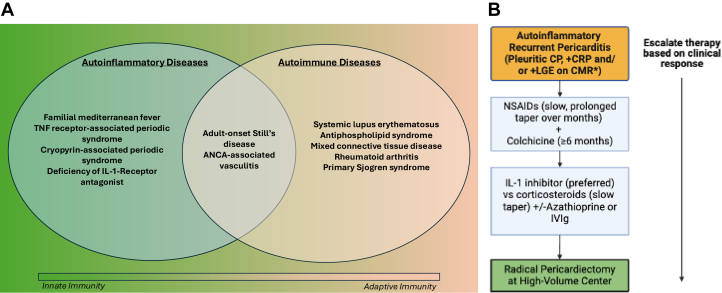
Spectrum of Autoinflammatory and Autoimmune Diseases and Treatment Algorithm for Autoinflammatory Recurrent Pericarditis (A)Venn diagram illustrating the spectrum of autoinflammatory and autoimmune diseases and the continuum between innate and adaptive immunity. (B) Stepwise treatment approach for autoinflammatory recurrent pericarditis. ∗Cardiac magnetic resonance imaging (CMR) plays a crucial role in imaging-guided treatment for RP, providing both diagnostic and prognostic insights. It can also help predict a patient’s response to therapy. ANCA = antineutrophil cytoplasmic autoantibody; CP = chest pain; CRP = C-reactive protein; IL = interleukin; IVIg = intravenous immunoglobulin; LGE = late gadolinium enhancement; NSAID = nonsteroidal anti-inflammatory drug; TNF = tumor necrosis factor.

We describe a case of refractory RP in which work-up revealed TRAPS as the etiology, with a significant clinical response upon switching from canakinumab (a human monoclonal immunoglobulin G1 antibody against IL-1β) to rilonacept (an IL-1α and IL-1β cytokine trap). TRAPS is an autosomal-dominant autoinflammatory disease caused by a mutation in the TNFRSF1A gene on chromosome 12p13.^4^ It is 1 of 4 monogenic autoinflammatory conditions associated with IL-1-mediated pericarditis (which are distinct from autoimmune diseases)^2^^,^^5^ (Figure 2A). Clinically, it presents as cyclic episodes of prolonged fevers (1-3 weeks) and inflammatory reactions in various body sites.^4^ Pericarditis is present in about 30% of individuals with TRAPS and is typically more responsive to corticosteroids than colchicine in this population.^6^ In an observational study of 131 patients, TNFRSF1A gene mutations were found in about 6% of patients with RP and were strongly correlated with a positive family history of pericarditis and/or recurrent fever syndrome, RP after the first year from the initial episode, failure to respond to colchicine, and the need for immunosuppressive agents.^7^

Imazio et al^8^ reported an observational study of 108 patients with RP, of whom 15% were found to have at least 1 variant associated with their disease. Gattorno et al^9^ developed evidence-based classification criteria on the basis of a large data set in the Eurofever registry to help identify TRAPS and distinguish it from other autoinflammatory conditions. According to these criteria, a diagnosis of TRAPS is highly suspected with a heterozygous pathogenic (or likely pathogenic) variant in TNFRSF1A identified by genetic testing in the context of at least 1 of the following clinical features: a flare lasting at least 7 days, myalgia, migratory rash, periorbital edema, or a suggestive family history. The investigators reported sensitivity of 94% to 100% and specificity of 95% to 100% using these criteria but emphasized that they are not intended for diagnostic purposes. In our case, there was high suspicion for TRAPS on the basis of molecular identification of the heterozygous variant and the duration of her fevers with RP. We consider genetic testing for RP on a case-by-case basis for the following scenarios: refractory incessant disease, RP with inability to wean off anti-inflammatory therapies, the presence of multisystem involvement (eg, unexplained or migratory rash, uveitis, serositis, and/or systemic amyloidosis with pericarditis), coexisting features suggestive of autoinflammatory syndromes (eg, cyclic fevers), or family history of pericarditis, serositis, or autoinflammatory disorders.

Last, radical pericardiectomy at centers of excellence is an option for patients with RP refractory or intolerant to medical therapy^10^ (Figure 2B).

## Conclusions

Refractory pericarditis in the context of TRAPS presents a significant clinical challenge because of its recurrent nature and complex underlying inflammatory mechanisms. Traditional therapies often fall short of providing sustained relief, highlighting the need for more targeted treatment options. The advent of IL-1 inhibitors offers a promising therapeutic avenue with good safety and efficacy profiles. This case underscores the importance of recognizing the potential for TRAPS in patients with RP and considering advanced biologic treatments and potentially radical pericardiectomy in refractory cases. RP is a rare disease, and there is a need to further understand its genetics and pathophysiology beyond monogenic autoinflammatory disease.

## Funding Support and Author Disclosures

Dr Al-Kazaz has received research grants and speaking honoraria from Kiniksa Pharmaceuticals. All other authors have reported that they have no relationships relevant to the contents of this paper to disclose.

## Appendix

Video 1 Parasternal Long-Axis View Demonstrating Mild Pericardial Thickening and a Lateral Pericardial Effusion Adjacent to the Left Ventricle Measuring 0.4 cm

Video 2 Apical 4-Chamber View Demonstrating an Apical Pericardial Effusion Measuring 0.8 cm
